# Structural investigation of methyl 3-(4-fluoro­benzo­yl)-7-methyl-2-phenyl­indolizine-1-carboxyl­ate, an inhibitory drug towards *Mycobacterium tuberculosis*

**DOI:** 10.1107/S2056989020003837

**Published:** 2020-03-20

**Authors:** Avantika Hasija, Subhrajyoti Bhandary, Katharigatta N. Venugopala, Sandeep Chandrashekharappa, Deepak Chopra

**Affiliations:** a Department of Chemistry, Indian Institute of Science Education and Research Bhopal, Bhauri, Bhopal 462066, India; b Department of Pharmaceutical Sciences, College of Clinical Pharmacy, King Faisal University, Al-Ahsa 31982, Kingdom of Saudi Arabia; c Department of Biotechnology and Food Technology, Durban University of Technology, Durban 4001, South Africa; d Institute for Stem Cell Biology and Regenerative Medicine, NCBS, TIFR, GKVK, Bellary Road, Bangalore 560 065, India

**Keywords:** crystal structure, anti-TB activity drug, inter­molecular inter­actions, Hirshfeld surface analysis, fingerprint plot

## Abstract

The title compound, C_24_H_18_FNO_3_, crystallizes in the monoclinic centrosymmetric space group *P2_1_/n* and its mol­ecular conformation is stabilized *via* C—H⋯O intra­molecular inter­actions. The supra­molecular network mainly comprises C—H⋯O, C—H⋯F and C—H⋯π inter­actions, which contribute towards the formation of the crystal structure. The different inter­molecular inter­actions have been further analysed *via* Hirshfeld surface analysis and fingerprint plots.

## Abstract 2

The structural analysis of a phenyl­indolizine-based drug, namely methyl 3-(4-flurobenzo­yl)-7-methyl-2-phenyl­indolizine-1-carboxyl­ate (I) was carried out; this drug shows an inhibitory action towards *mycobacterium tuberculosis*. The inter­molecular inter­actions at play were characterized *via* Hirshfeld surface analysis and fingerprint plots, highlighting the evident role of C—H⋯O, C—H⋯F and C—H⋯π inter­actions in the formation of the observed crystal structure.

## Chemical context   

Indolizine represents an inter­esting heterocyclic scaffold in which the nitro­gen atom belongs to both of the fused six- and five-membered rings. It is a well-known pharmacophore endowed with various promising pharmacological properties. For instance, indolizines have been found to exhibit analgesic (Vaught *et al.*, 1990 ▸), anti­cancer (Butler, 2008 ▸; Sandeep *et al.*, 2016*a* ▸,*b* ▸), anti­diabetic (Mederski *et al.*, 2012 ▸), anti­histaminic (Cingolani *et al.*, 1990 ▸), anti-microbial (Hazra *et al.*, 2011 ▸) and anti­viral (Mishra & Tiwari, 2011 ▸) activity. It has also been found to act as cyclo-oxygenase (COX-2) inhibitor (Chandrashekharappa *et al.*, 2018*b* ▸) and to have larvicidal activity against *Anopheles arabiensis* (Chandrashekharappa *et al.*, 2018*a* ▸).
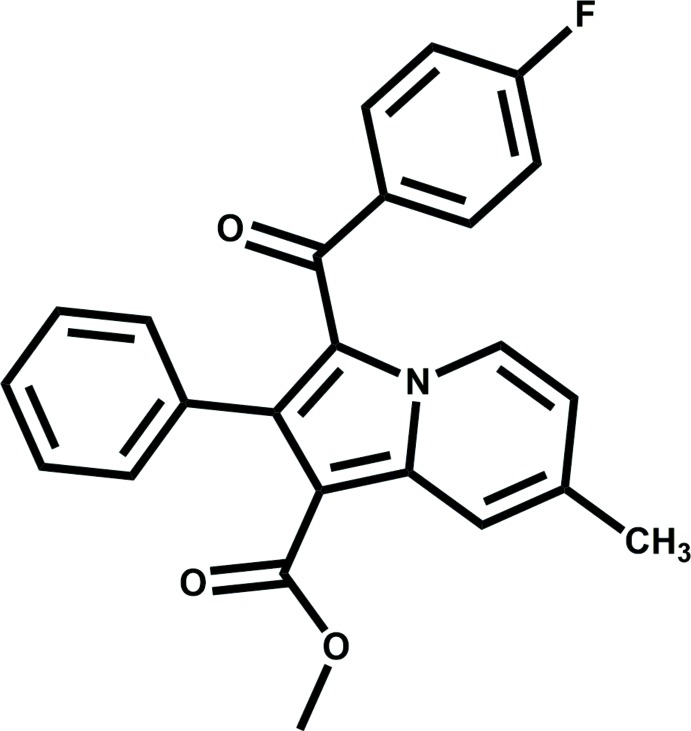


The title compound, comprising a substituted indolizine unit, displays a modest activity against susceptible H37Rv strains of *Mycobacterium tuberculosis* (Venugopala *et al.*, 2019 ▸). Besides the tremendous scope of the pharmacological studies on indolizine-based compounds, the substitution of fluorine on the benzoyl ring, the presence of flexible moieties and of competitive hydrogen-bond acceptors (namely, oxygen O2 in the ester group at C6 and O3 in the carbonyl group at C8) make the structural study of the title compound of extreme relevance. In addition, it is of importance to observe the cooperative inter­play of weak inter­actions that contribute towards the consolidation of the crystal lattice. In the present paper, we report the mol­ecular and crystal structure of the title compound, highlighting its mol­ecular conformation and analysing the different inter­molecular inter­actions *via* Hirshfeld surface analysis and fingerprint plots.

## Structural commentary   

**Figure 1:**
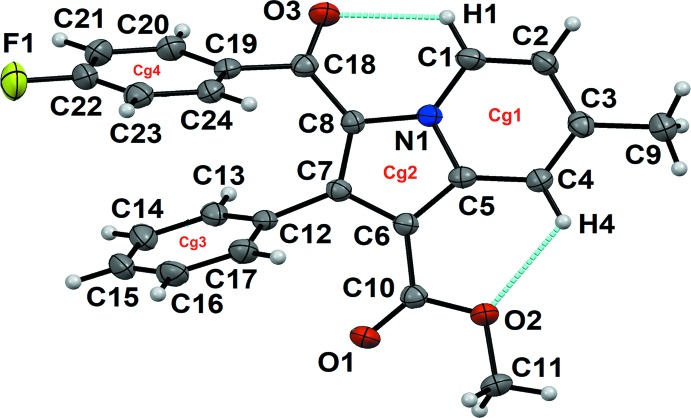
Ellipsoid plot of the title compound drawn with 50% probability ellipsoids. Dotted lines indicate intra­molecular C—H⋯O inter­actions. *Cg*1, *Cg*3 and *Cg*4 represent the centroids of the six-membered rings N1/C1–C5, C12–C17 and C18/O3/C19–C24/F1, respectively, while *Cg*2 represents the five-membered ring N1/C5–C8.

**Figure 2:**
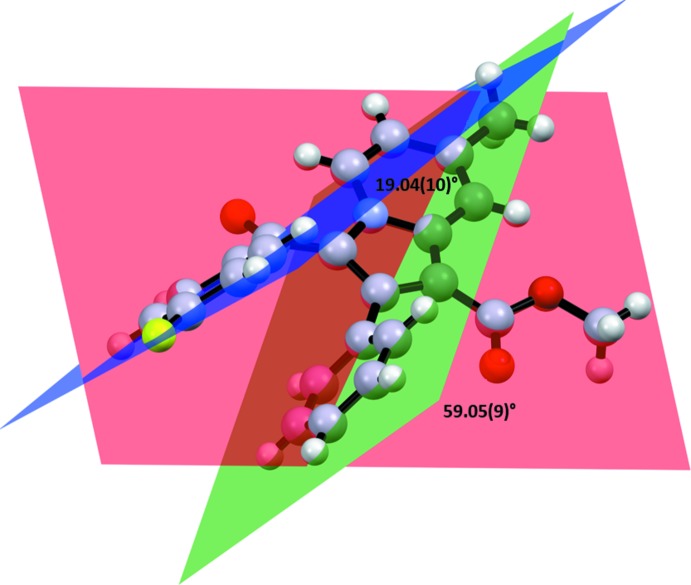
Dihedral angles between the mean plane passing through the C12–C17 ring (green) and the C18/O3/C19–C24/F1 ring (blue) and through the indolizine skeleton (red).

The title compound crystallizes in the centrosymmetric monoclinic *P2_1_/n* space group. The mol­ecular structure comprises one methyl­indolizine heterocyclic moiety (N1/C1–C9) consisting of fused six- and five-membered rings (N1/C1–C5, centroid *Cg*1 and N1/C5–C8, centroid *Cg*2). The heterocycle is substituted at the carbon atoms C6, C7 and C8 with a meth­oxy carbonyl group, a phenyl ring (C12–C17, centroid *Cg*3), and a fluoro­benzoyl ring [C18/O3/C19–C24/F1, centroid *Cg*4], respectively (Fig. 1 ▸). The mol­ecular structure possesses three conformational degrees of freedom due to the free rotation with respect to the C6—C10, C7—C12, and C8—C18 single bonds. The mol­ecular conformation is stabilized by the presence of intra­molecular C1—H1⋯O3 [C1⋯O3 = 2.853 (3) Å] and C4—H4⋯O2 [C4⋯O2 = 2.927 (2) Å] inter­actions (Table 1 ▸) and by π–π stacking [*Cg*3⋯*Cg*4 = 3.5084 (13) Å]. The dihedral angle between the mean plane through ring *Cg*3 (coloured in green in Fig. 2 ▸) and the mean plane of the indolizine skeleton (coloured in red) is 59.05 (9)°, while the dihedral angle between the mean plane through the phenyl ring and that through the fluoro­benzoyl ring (coloured in blue) is as small as 19.04 (10),° showing the nearly parallel position of the rings. The torsion angles N1—C8—C18—C19 and C8—C18—C19—C24 are −161.74 (19) and 46.2 (3)°, respectively.

## Supra­molecular features   

**Figure 3:**
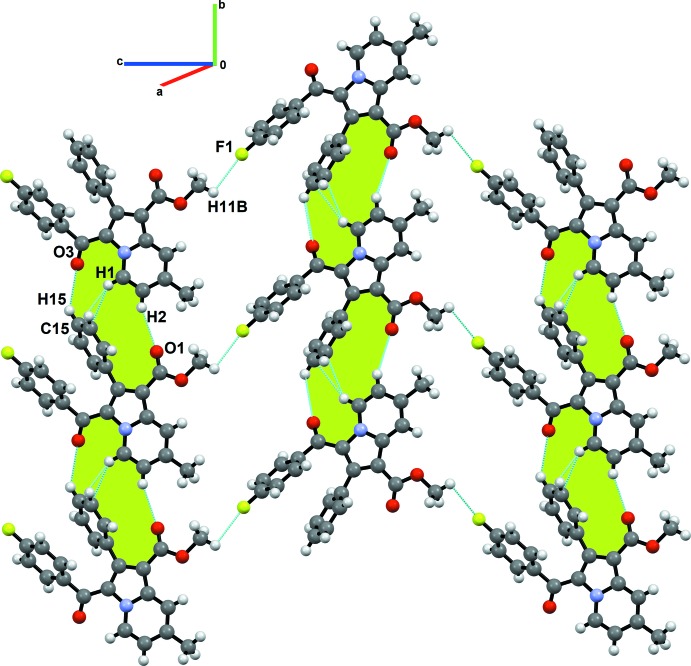
Crystal packing of title compound showing the formation of mol­ecular sheets parallel to the *bc* plane *via* C—H⋯O, C—H⋯π and C—H⋯F inter­actions.

**Figure 4:**
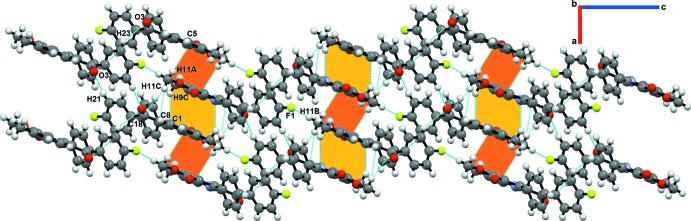
Stacking of mol­ecular sheets along the *a*-axis direction, primarily *via* C—H⋯π and C—H⋯F inter­actions, resulting in a layered supra­molecular architecture.

The list of all intra- and inter­molecular inter­actions along with their geometrical parameters have been reported in Table 1 ▸. The inter­actions included for investigation are based on the distance criteria of vdW + 0.4 Å (Dance, 2003 ▸). In the crystal, the mol­ecules are primarily assembled through concomitant C2/15—H2/15⋯O1^ii^/O3^iii^ inter­actions [C2⋯O1^ii^ = 3.531 (4) Å, 157°; C15⋯O3^iii^ = 3.519 (4) Å, 137°; symmetry codes: (ii) *x*, *y* − 1, *z*; (iii) *x*, *y* + 1, *z*] and C1—H1⋯π(C15)^ii^ [C1⋯C15 = 3.6064 (3) Å, 152°], forming ribbons along the [010] direction, as shown by the green shading in Fig. 3 ▸. Two adjacent ribbons are connected to each other *via* C11—H11*B*⋯F1^v^ [C11⋯F1 = 3.0585 (3) Å, 104°; symmetry code: (v) *x* − 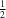, −*y* + 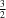, *z* − 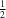] (Fig. 3 ▸) and C21—H21⋯O3^i^ [C21⋯O3 = 3.399 (3) Å, 149°; symmetry code: (i) −*x* + 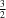, *y* + 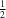, −*z* + 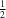] (Fig. 4 ▸) inter­actions in a zigzag fashion along [001], resulting in the formation of a mol­ecular sheet parallel to the *ac* plane. Analogous C—H⋯F inter­actions have been investigated, showing that where the angularity is in the range 90 to 140°, the σ-hole on fluorine is directed towards the electron density of the C—H bond (Hathwar *et al.*, 2020 ▸), underlining the importance of inter­actions with low angularity. The mol­ecular sheets are closely stacked along the *a*-axis direction *via* weak inter­actions such as C9—H9*C*⋯π(C1) [C9⋯C1^vii^ = 3.7431 (5) Å; symmetry code: (vii) −*x* + 1, −*y*, −*z*], C11—H11*A*⋯π(C5) [C11⋯C5^iv^ = 3.4906 (4) Å; symmetry code: (iv) −*x*, −*y* + 1, −*z*], C11—H11*C*⋯π(C8) [C11⋯C8^viii^ = 3.6590 (5) Å; symmetry code: (viii) −*x* + 1, −*y* + 1, −*z*] (Fig. 4 ▸), giving rise to a layered supra­molecular structure. From this analysis, it can be stated that the formation of the crystal structure is mainly governed by several C—H⋯O and C—H⋯π inter­actions, while the C—H⋯F inter­actions play a secondary but supporting role in its overall consolidation.

## Database survey   

A search for the 2-phenyl­indolizine skeleton in the CSD (version 5.40, update of August 2019; Groom *et al.*, 2016 ▸) was carried out. Out of the 39 hits for unsubstituted phenyl rings attached to indolizine, the majority of entries gave reports of varied synthetic procedures and methodologies to obtain these compounds, underlining their importance. The near-infrared emissive properties of KIVLIN, KIVLOT, KIVLUZ (Gayton *et al.*, 2019 ▸) and KENFAN (McNamara *et al.*, 2017 ▸) have also been reported.

Structural details of compounds such as CAJTAI (Aslanov *et al.*, 1983 ▸), EMUTOV (Liu, *et al.*, 2003 ▸), FEDQAH (Liu, *et al.*, 2005 ▸), GIYLOP (Sonnenschein & Schneider, 1997 ▸), ODEFIN (Qian *et al.*, 2006 ▸), PNOIZA, PNOIZB, PNOIZE, PNOIZF (Tafeenkov & Aslanov, 1980 ▸), ROLKIM (Tafeenkov & Au, 1996 ▸) and TIGXOX (Liu, *et al.*, 2007 ▸) have also been deposited. Almost all of these mol­ecules are substituted at the C8 position with electron-withdrawing substituents such as –COMe, –CH_2_CN, –CN, –N=O, –CH=C(Ph)(CN), *etc*.

In particular, the papers reporting TIGXOX (Liu *et al.*, 2007 ▸), FEDQAH (Liu *et al.*, 2005 ▸) and ODEFIN (Qian *et al.*, 2006 ▸) discuss the structural features of mol­ecules comprising the 2-phenyl indolizine skeleton, showing high fluorescent efficiency. In these reports, the respective dihedral angles between the mean plane of the indolizine skeleton and the plane of the phenyl ring are *ca* 53, 39 and 49 and 45°, comparable to that reported in the title compound.

## Hirshfeld surface analysis and fingerprint plots   

**Figure 5:**
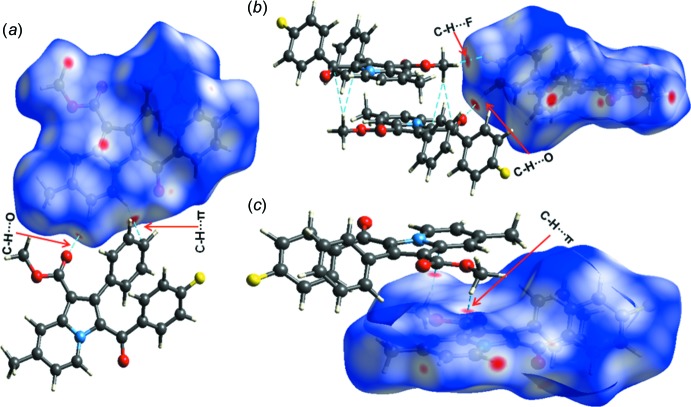
The Hirshfeld surface of title compound mapped over *d*_norm_. Dashed lines indicate hydrogen bonds.

**Figure 6:**
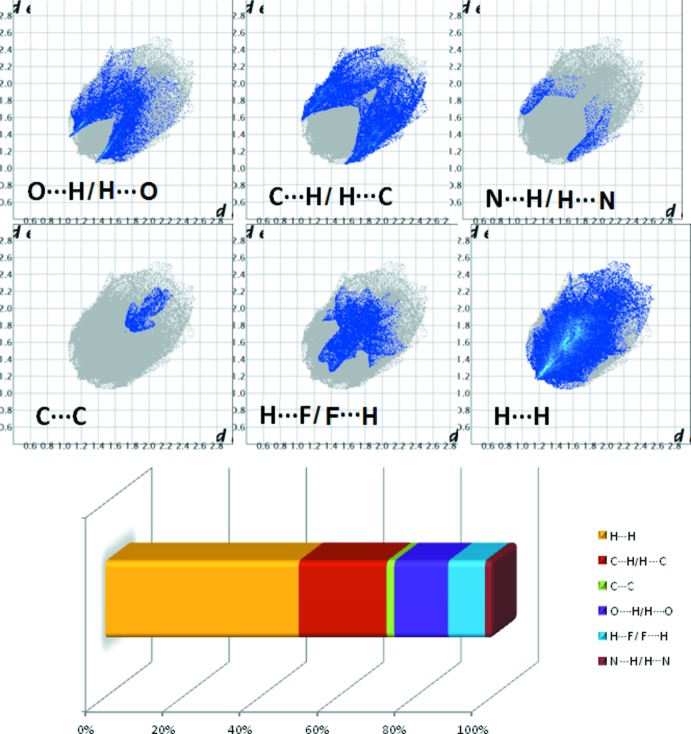
The fingerprint plots of the title compound showing the different contributions deriving from the O⋯H/H⋯O, N⋯H/H⋯N, C⋯H/H⋯C, H⋯F/F⋯H, C⋯C and H⋯H contacts.

The significance of the cumulative effect of the inter­actions involved in the crystal structure can be visualized qualitatively through Hirshfeld surface analysis (Spackman *et al.*, 2009 ▸). The Hirshfeld surfaces and the two-dimensional fingerprint plots were calculated using *CrystalExplorer* (Version 17.5; Wolff *et al.*, 2012 ▸) and are shown in Figs. 5 ▸ and 6 ▸, respectively. The red spots on the HS surface illustrate the presence of supra­molecular inter­actions such as C—H⋯O, C—H⋯π and C—H⋯F whereas the blue regions indicate the lack of contact distances shorter than the sum of the van der Waals radii. The fingerprint plots represent the individual contributions of the different inter­actions. Fig. 6 ▸ shows that the major contribution comes from H⋯H (47.1%), O⋯H/H⋯O (13.1%), C⋯H/ H⋯C (21.4%), H⋯F/F⋯H (9.0%), C⋯C (1.9%) and N⋯H/H⋯N (1.7%) contacts. The relatively high percentage of C⋯H/H⋯C contacts indicates how the contribution of all of the C—H⋯π inter­actions plays an important role in consolidating the crystal packing.

## Synthesis and crystallization   

**Figure 7:**
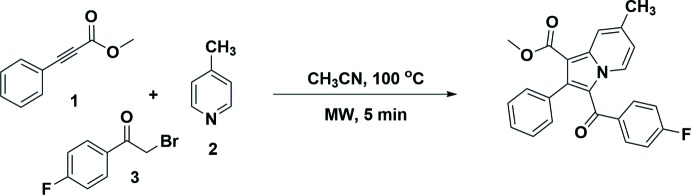
The reaction scheme for the synthesis of the title compound.

All chemicals were obtained from Sigma–Aldrich and used without further purification. A mixture of methyl 3-phenyl­propiolate (**1**) (160 mg, 1 mmol), 4-methyl­pyridine (**2**) (93 mg, 1 mmol), 2-bromo-1-(4-fluoro­phen­yl)ethan-1-one (**3**) (217 mg, 1 mmol), and tri­ethyl­amine (0.101 mg, 1 mmol) in 4.5 mL of aceto­nitrile were added to a 10 mL microwave tube under a nitro­gen atmosphere (Fig. 7 ▸). A microwave initiator was used to irradiate the reaction mixture at 373 K for about 5 min. The reaction was monitored *via* TLC. The solvent was then removed under reduced pressure, the crude residue was diluted with water and the aqueous layer was extracted twice with ethyl acetate, and the combined organic solvent was washed with a brine solution. The organic layer was removed under reduced pressure and the remaining residue was subjected to column chromatography using 60–120 mesh silica gel with an ethyl acetate and hexane solvent system to afford 0.3414 g (88% yield) of the title compound (Venugopala *et al.*, 2019 ▸). Suitable single crystals of the compound were grown by the slow evaporation of acetone at ambient conditions.

## Refinement   

Crystal data, data collection and structure refinement details are summarized in Table 2 ▸. The hydrogen atoms were placed in idealized positions and refined using a riding model with *U*_iso_(H) =1.2*U*_eq_(C) or 1.5*U*_eq_(C-meth­yl).

## Supplementary Material

Crystal structure: contains datablock(s) I. DOI: 10.1107/S2056989020003837/xi2021sup1.cif

Structure factors: contains datablock(s) I. DOI: 10.1107/S2056989020003837/xi2021Isup2.hkl

Supporting information file. DOI: 10.1107/S2056989020003837/xi2021Isup3.cml

CCDC reference: 1865697

Additional supporting information:  crystallographic information; 3D view; checkCIF report

## Figures and Tables

**Table 1:** Hydrogen-bond geometry (Å, °)

*D*—H⋯*A*	*D*—H	H⋯*A*	*D*⋯*A*	*D*—H⋯*A*
C1—H1⋯O3	0.95	2.26	2.853 (3)	120
C4—H4⋯O2	0.95	2.38	2.927 (2)	116
C21—H21⋯O3^i^	0.95	2.54	3.399 (3)	149
C2—H2⋯O1^ii^	0.95	2.63	3.531 (4)	157
C15—H15⋯O3^iii^	0.95	2.76	3.519 (4)	137
C1—H1⋯C15^ii^	0.95	2.74	3.6064 (3)	152
C11—H11*A*⋯C5^iv^	0.98	2.74	3.4906 (1)	133
C11—H11*B*⋯F1^v^	0.98	2.67	3.0585 (3)	104
C23—H23⋯O3^vi^	0.95	2.67	3.4875 (3)	143

Symmetry codes: (i) 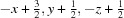; (ii) 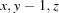; (iii) 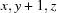; (iv) 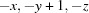; (v) 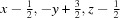; (vi) 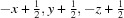.

**Table 2:** Experimental details

Crystal data	
Chemical formula	C_24_H_18_FNO_3_
*M*_r_	387.39
Crystal system, space group	Monoclinic, *P*2_1_/*n*
Temperature (K)	173
*a*, *b*, *c* (Å)	7.3246 (11), 9.8460 (13), 25.837 (4)
β (°)	93.318 (3)
*V* (Å^3^)	1860.2 (5)
*Z*	4
Radiation type	Mo *K*α
μ (mm^−1^)	0.10
Crystal size (mm)	0.32 × 0.18 × 0.04

Data collection	
Diffractometer	Bruker Kappa Duo APEXII
Absorption correction	Multi-scan (*SADABS*; Bruker, 2008 ▸)
*T*_min_, *T*_max_	0.855, 1.000
No. of measured, independent and observed [*I* > 2σ(*I*)] reflections	27523, 4296, 2641
*R*_int_	0.090
(sin θ/λ)_max_ (Å^−1^)	0.652

Refinement	
*R*[*F* ^2^ > 2σ(*F* ^2^)], *wR*(*F* ^2^), *S*	0.050, 0.131, 1.00
No. of reflections	4296
No. of parameters	265
H-atom treatment	H-atom parameters constrained
Δρ_max_, Δρ_min_ (e Å^−3^)	0.29, −0.30

Computer programs: *APEX2* (Bruker, 2012 ▸), *SAINT* (Bruker, 2008 ▸), *SHELXS97* (Sheldrick, 2008 ▸), *X-SEED* (Barbour, 2001 ▸), *Mercury* (Macrae *et al.*, 2020 ▸), *SHELXL2014* (Sheldrick, 2015 ▸) and *PLATON* (Spek, 2020 ▸).

## supplementary crystallographic information

### Crystal data

**Table:**

C_24_H_18_FNO_3_	*F*(000) = 808
*M**_r_* = 387.39	*D*_x_ = 1.383 Mg m^−^^3^
Monoclinic, *P*2_1_/*n*	Mo *K*α radiation, λ = 0.71073 Å
*a* = 7.3246 (11) Å	Cell parameters from 27523 reflections
*b* = 9.8460 (13) Å	θ = 2.2–27.6°
*c* = 25.837 (4) Å	µ = 0.10 mm^−^^1^
β = 93.318 (3)°	*T* = 173 K
*V* = 1860.2 (5) Å^3^	Block, yellow
*Z* = 4	0.32 × 0.18 × 0.04 mm

### Data collection

**Table:**

Bruker Kappa Duo APEXII diffractometer	2641 reflections with *I* > 2σ(*I*)
Radiation source: fine-focus sealed tube	*R*_int_ = 0.090
0.5° φ scans and ω scans	θ_max_ = 27.6°, θ_min_ = 2.2°
Absorption correction: multi-scan (SADABS; Bruker, 2008)	*h* = −9→9
*T*_min_ = 0.855, *T*_max_ = 1.000	*k* = −12→12
27523 measured reflections	*l* = −33→33
4296 independent reflections	

### Refinement

**Table:**

Refinement on *F*^2^	Secondary atom site location: difference Fourier map
Least-squares matrix: full	Hydrogen site location: inferred from neighbouring sites
*R*[*F*^2^ > 2σ(*F*^2^)] = 0.050	H-atom parameters constrained
*wR*(*F*^2^) = 0.131	*w* = 1/[σ^2^(*F*_o_^2^) + (0.0555*P*)^2^ + 0.455*P*] where *P* = (*F*_o_^2^ + 2*F*_c_^2^)/3
*S* = 1.00	(Δ/σ)_max_ < 0.001
4296 reflections	Δρ_max_ = 0.29 e Å^−^^3^
265 parameters	Δρ_min_ = −0.30 e Å^−^^3^
0 restraints	Extinction correction: SHELXL-2014/7 (Sheldrick 2015, Fc^*^=kFc[1+0.001xFc^2^λ^3^/sin(2θ)]^-1/4^
Primary atom site location: structure-invariant direct methods	Extinction coefficient: 0.0044 (8)

### Special details

**Table:**

Geometry. All esds (except the esd in the dihedral angle between two l.s. planes) are estimated using the full covariance matrix. The cell esds are taken into account individually in the estimation of esds in distances, angles and torsion angles; correlations between esds in cell parameters are only used when they are defined by crystal symmetry. An approximate (isotropic) treatment of cell esds is used for estimating esds involving l.s. planes.

### Fractional atomic coordinates and isotropic or equivalent isotropic displacement parameters (Å^2^)

**Table:**

	*x*	*y*	*z*	*U*_iso_*/*U*_eq_	
F1	0.4480 (2)	0.70181 (14)	0.32107 (5)	0.0563 (4)	
O1	0.2787 (2)	0.69998 (15)	0.00174 (6)	0.0381 (4)	
O2	0.1668 (2)	0.53237 (14)	−0.04937 (5)	0.0302 (4)	
O3	0.5350 (2)	0.21447 (15)	0.16777 (5)	0.0336 (4)	
N1	0.3494 (2)	0.26669 (16)	0.06884 (6)	0.0230 (4)	
C1	0.3588 (3)	0.1276 (2)	0.07210 (8)	0.0293 (5)	
H1	0.4025	0.0857	0.1035	0.035*	
C2	0.3057 (3)	0.0502 (2)	0.03060 (8)	0.0294 (5)	
H2	0.3148	−0.0459	0.0330	0.035*	
C3	0.2368 (3)	0.1106 (2)	−0.01642 (8)	0.0269 (5)	
C4	0.2282 (3)	0.2488 (2)	−0.01917 (7)	0.0252 (5)	
H4	0.1830	0.2906	−0.0505	0.030*	
C5	0.2850 (3)	0.3307 (2)	0.02346 (7)	0.0232 (4)	
C6	0.2913 (3)	0.4722 (2)	0.03281 (7)	0.0232 (4)	
C7	0.3565 (3)	0.4924 (2)	0.08437 (7)	0.0225 (4)	
C8	0.3910 (3)	0.3647 (2)	0.10738 (7)	0.0229 (4)	
C9	0.1754 (3)	0.0225 (2)	−0.06142 (8)	0.0356 (5)	
H9A	0.1001	0.0760	−0.0865	0.053*	
H9B	0.1035	−0.0537	−0.0491	0.053*	
H9C	0.2826	−0.0125	−0.0781	0.053*	
C10	0.2478 (3)	0.5806 (2)	−0.00470 (7)	0.0248 (5)	
C11	0.1182 (3)	0.6340 (2)	−0.08794 (8)	0.0315 (5)	
H11A	0.0323	0.6988	−0.0739	0.047*	
H11B	0.0606	0.5902	−0.1188	0.047*	
H11C	0.2285	0.6822	−0.0974	0.047*	
C12	0.3881 (3)	0.6250 (2)	0.11063 (7)	0.0250 (5)	
C13	0.5610 (3)	0.6581 (2)	0.13180 (7)	0.0287 (5)	
H13	0.6610	0.5989	0.1269	0.034*	
C14	0.5889 (3)	0.7767 (2)	0.15993 (8)	0.0363 (6)	
H14	0.7076	0.7984	0.1743	0.044*	
C15	0.4445 (4)	0.8634 (2)	0.16707 (8)	0.0387 (6)	
H15	0.4631	0.9443	0.1867	0.046*	
C16	0.2724 (4)	0.8320 (2)	0.14543 (8)	0.0390 (6)	
H16	0.1729	0.8918	0.1502	0.047*	
C17	0.2443 (3)	0.7144 (2)	0.11693 (8)	0.0326 (5)	
H17	0.1263	0.6945	0.1016	0.039*	
C18	0.4633 (3)	0.3265 (2)	0.15900 (7)	0.0253 (5)	
C19	0.4534 (3)	0.4247 (2)	0.20269 (7)	0.0257 (5)	
C20	0.6087 (3)	0.4441 (2)	0.23563 (8)	0.0324 (5)	
H20	0.7157	0.3924	0.2307	0.039*	
C21	0.6082 (3)	0.5381 (2)	0.27542 (8)	0.0385 (6)	
H21	0.7144	0.5534	0.2975	0.046*	
C22	0.4491 (4)	0.6086 (2)	0.28191 (8)	0.0374 (6)	
C23	0.2923 (3)	0.5899 (2)	0.25163 (8)	0.0328 (5)	
H23	0.1843	0.6393	0.2579	0.039*	
C24	0.2955 (3)	0.4967 (2)	0.21143 (8)	0.0283 (5)	
H24	0.1883	0.4820	0.1897	0.034*	

### Atomic displacement parameters (Å^2^)

**Table:**

	*U*^11^	*U*^22^	*U*^33^	*U*^12^	*U*^13^	*U*^23^
F1	0.0812 (12)	0.0445 (9)	0.0415 (8)	0.0051 (8)	−0.0116 (7)	−0.0201 (7)
O1	0.0596 (11)	0.0203 (8)	0.0333 (8)	−0.0034 (7)	−0.0062 (7)	0.0057 (7)
O2	0.0413 (9)	0.0238 (8)	0.0248 (7)	0.0018 (7)	−0.0048 (6)	0.0033 (6)
O3	0.0420 (9)	0.0259 (8)	0.0323 (8)	0.0073 (7)	−0.0035 (7)	0.0032 (7)
N1	0.0271 (10)	0.0201 (9)	0.0219 (8)	0.0005 (7)	0.0025 (7)	0.0018 (7)
C1	0.0363 (13)	0.0213 (11)	0.0304 (11)	0.0029 (9)	0.0019 (9)	0.0052 (9)
C2	0.0369 (13)	0.0192 (11)	0.0324 (11)	0.0007 (9)	0.0036 (9)	−0.0004 (9)
C3	0.0282 (11)	0.0251 (12)	0.0280 (11)	−0.0045 (9)	0.0057 (9)	−0.0019 (9)
C4	0.0282 (11)	0.0248 (11)	0.0228 (10)	−0.0010 (9)	0.0020 (8)	0.0008 (8)
C5	0.0235 (10)	0.0234 (11)	0.0228 (10)	0.0005 (8)	0.0032 (8)	0.0033 (8)
C6	0.0255 (11)	0.0214 (10)	0.0228 (10)	0.0001 (8)	0.0020 (8)	0.0010 (8)
C7	0.0239 (11)	0.0206 (10)	0.0233 (10)	0.0014 (8)	0.0028 (8)	0.0005 (8)
C8	0.0260 (11)	0.0207 (10)	0.0219 (9)	0.0001 (8)	0.0021 (8)	−0.0014 (8)
C9	0.0460 (14)	0.0281 (12)	0.0326 (12)	−0.0038 (10)	0.0005 (10)	−0.0039 (10)
C10	0.0268 (11)	0.0243 (11)	0.0237 (10)	0.0000 (9)	0.0033 (8)	0.0015 (9)
C11	0.0382 (13)	0.0302 (12)	0.0256 (10)	0.0028 (10)	−0.0036 (9)	0.0077 (9)
C12	0.0373 (12)	0.0183 (10)	0.0197 (9)	−0.0001 (9)	0.0028 (9)	0.0029 (8)
C13	0.0375 (12)	0.0237 (11)	0.0253 (10)	−0.0035 (9)	0.0056 (9)	0.0008 (9)
C14	0.0494 (15)	0.0283 (12)	0.0311 (12)	−0.0106 (11)	0.0033 (11)	−0.0014 (10)
C15	0.0653 (17)	0.0199 (12)	0.0311 (12)	−0.0025 (11)	0.0026 (11)	−0.0030 (10)
C16	0.0589 (16)	0.0251 (12)	0.0333 (12)	0.0136 (11)	0.0062 (11)	0.0012 (10)
C17	0.0407 (13)	0.0267 (12)	0.0301 (11)	0.0059 (10)	−0.0009 (10)	0.0018 (9)
C18	0.0254 (11)	0.0259 (11)	0.0247 (10)	−0.0020 (9)	0.0026 (8)	0.0024 (9)
C19	0.0344 (12)	0.0223 (11)	0.0202 (9)	−0.0025 (9)	0.0000 (9)	0.0045 (8)
C20	0.0371 (13)	0.0289 (12)	0.0305 (11)	0.0013 (10)	−0.0046 (10)	0.0022 (10)
C21	0.0498 (16)	0.0324 (13)	0.0316 (12)	−0.0036 (11)	−0.0130 (11)	0.0016 (10)
C22	0.0614 (17)	0.0235 (12)	0.0268 (11)	−0.0004 (11)	−0.0024 (11)	−0.0027 (9)
C23	0.0420 (14)	0.0298 (12)	0.0269 (11)	0.0020 (10)	0.0058 (10)	0.0018 (9)
C24	0.0346 (12)	0.0274 (12)	0.0228 (10)	−0.0028 (9)	0.0010 (9)	0.0018 (9)

### Geometric parameters (Å, º)

**Table:**

F1—C22	1.367 (2)	C11—H11A	0.9800
O1—C10	1.206 (2)	C11—H11B	0.9800
O2—C10	1.353 (2)	C11—H11C	0.9800
O2—C11	1.442 (2)	C12—C13	1.389 (3)
O3—C18	1.236 (2)	C12—C17	1.389 (3)
N1—C1	1.373 (3)	C13—C14	1.384 (3)
N1—C5	1.389 (2)	C13—H13	0.9500
N1—C8	1.408 (2)	C14—C15	1.380 (3)
C1—C2	1.354 (3)	C14—H14	0.9500
C1—H1	0.9500	C15—C16	1.384 (3)
C2—C3	1.419 (3)	C15—H15	0.9500
C2—H2	0.9500	C16—C17	1.382 (3)
C3—C4	1.364 (3)	C16—H16	0.9500
C3—C9	1.499 (3)	C17—H17	0.9500
C4—C5	1.409 (3)	C18—C19	1.491 (3)
C4—H4	0.9500	C19—C24	1.387 (3)
C5—C6	1.414 (3)	C19—C20	1.393 (3)
C6—C7	1.403 (3)	C20—C21	1.383 (3)
C6—C10	1.465 (3)	C20—H20	0.9500
C7—C8	1.407 (3)	C21—C22	1.375 (3)
C7—C12	1.483 (3)	C21—H21	0.9500
C8—C18	1.456 (3)	C22—C23	1.364 (3)
C9—H9A	0.9800	C23—C24	1.388 (3)
C9—H9B	0.9800	C23—H23	0.9500
C9—H9C	0.9800	C24—H24	0.9500
			
C10—O2—C11	115.09 (16)	H11A—C11—H11C	109.5
C1—N1—C5	121.17 (17)	H11B—C11—H11C	109.5
C1—N1—C8	129.22 (17)	C13—C12—C17	119.06 (19)
C5—N1—C8	109.56 (16)	C13—C12—C7	120.09 (18)
C2—C1—N1	120.09 (19)	C17—C12—C7	120.76 (19)
C2—C1—H1	120.0	C14—C13—C12	120.5 (2)
N1—C1—H1	120.0	C14—C13—H13	119.7
C1—C2—C3	120.92 (19)	C12—C13—H13	119.7
C1—C2—H2	119.5	C15—C14—C13	120.1 (2)
C3—C2—H2	119.5	C15—C14—H14	120.0
C4—C3—C2	118.41 (18)	C13—C14—H14	120.0
C4—C3—C9	121.74 (19)	C14—C15—C16	119.7 (2)
C2—C3—C9	119.85 (18)	C14—C15—H15	120.2
C3—C4—C5	121.33 (19)	C16—C15—H15	120.2
C3—C4—H4	119.3	C17—C16—C15	120.4 (2)
C5—C4—H4	119.3	C17—C16—H16	119.8
N1—C5—C4	118.07 (18)	C15—C16—H16	119.8
N1—C5—C6	107.26 (16)	C16—C17—C12	120.2 (2)
C4—C5—C6	134.66 (18)	C16—C17—H17	119.9
C7—C6—C5	107.94 (17)	C12—C17—H17	119.9
C7—C6—C10	125.01 (18)	O3—C18—C8	121.78 (18)
C5—C6—C10	126.97 (17)	O3—C18—C19	118.57 (17)
C6—C7—C8	108.49 (17)	C8—C18—C19	119.64 (17)
C6—C7—C12	126.51 (17)	C24—C19—C20	119.31 (19)
C8—C7—C12	124.99 (17)	C24—C19—C18	122.16 (18)
C7—C8—N1	106.72 (16)	C20—C19—C18	118.54 (19)
C7—C8—C18	131.69 (18)	C21—C20—C19	120.6 (2)
N1—C8—C18	121.52 (17)	C21—C20—H20	119.7
C3—C9—H9A	109.5	C19—C20—H20	119.7
C3—C9—H9B	109.5	C22—C21—C20	117.8 (2)
H9A—C9—H9B	109.5	C22—C21—H21	121.1
C3—C9—H9C	109.5	C20—C21—H21	121.1
H9A—C9—H9C	109.5	C23—C22—F1	118.3 (2)
H9B—C9—H9C	109.5	C23—C22—C21	123.6 (2)
O1—C10—O2	121.96 (18)	F1—C22—C21	118.1 (2)
O1—C10—C6	125.95 (18)	C22—C23—C24	117.9 (2)
O2—C10—C6	112.09 (17)	C22—C23—H23	121.0
O2—C11—H11A	109.5	C24—C23—H23	121.0
O2—C11—H11B	109.5	C19—C24—C23	120.7 (2)
H11A—C11—H11B	109.5	C19—C24—H24	119.6
O2—C11—H11C	109.5	C23—C24—H24	119.6
			
C5—N1—C1—C2	0.4 (3)	C7—C6—C10—O2	−172.44 (18)
C8—N1—C1—C2	177.53 (19)	C5—C6—C10—O2	11.1 (3)
N1—C1—C2—C3	−1.2 (3)	C6—C7—C12—C13	−121.3 (2)
C1—C2—C3—C4	1.2 (3)	C8—C7—C12—C13	57.4 (3)
C1—C2—C3—C9	−178.8 (2)	C6—C7—C12—C17	62.2 (3)
C2—C3—C4—C5	−0.4 (3)	C8—C7—C12—C17	−119.0 (2)
C9—C3—C4—C5	179.56 (19)	C17—C12—C13—C14	1.7 (3)
C1—N1—C5—C4	0.4 (3)	C7—C12—C13—C14	−174.86 (18)
C8—N1—C5—C4	−177.26 (17)	C12—C13—C14—C15	−0.2 (3)
C1—N1—C5—C6	179.55 (18)	C13—C14—C15—C16	−0.8 (3)
C8—N1—C5—C6	1.9 (2)	C14—C15—C16—C17	0.2 (3)
C3—C4—C5—N1	−0.4 (3)	C15—C16—C17—C12	1.3 (3)
C3—C4—C5—C6	−179.2 (2)	C13—C12—C17—C16	−2.2 (3)
N1—C5—C6—C7	−1.1 (2)	C7—C12—C17—C16	174.28 (19)
C4—C5—C6—C7	177.8 (2)	C7—C8—C18—O3	−157.8 (2)
N1—C5—C6—C10	175.82 (18)	N1—C8—C18—O3	19.0 (3)
C4—C5—C6—C10	−5.3 (4)	C7—C8—C18—C19	21.5 (3)
C5—C6—C7—C8	0.0 (2)	N1—C8—C18—C19	−161.76 (18)
C10—C6—C7—C8	−177.05 (19)	O3—C18—C19—C24	−134.5 (2)
C5—C6—C7—C12	178.88 (19)	C8—C18—C19—C24	46.2 (3)
C10—C6—C7—C12	1.9 (3)	O3—C18—C19—C20	45.5 (3)
C6—C7—C8—N1	1.2 (2)	C8—C18—C19—C20	−133.8 (2)
C12—C7—C8—N1	−177.78 (18)	C24—C19—C20—C21	−2.6 (3)
C6—C7—C8—C18	178.3 (2)	C18—C19—C20—C21	177.43 (19)
C12—C7—C8—C18	−0.7 (3)	C19—C20—C21—C22	1.4 (3)
C1—N1—C8—C7	−179.3 (2)	C20—C21—C22—C23	0.6 (3)
C5—N1—C8—C7	−1.9 (2)	C20—C21—C22—F1	−179.8 (2)
C1—N1—C8—C18	3.2 (3)	F1—C22—C23—C24	178.97 (19)
C5—N1—C8—C18	−179.35 (17)	C21—C22—C23—C24	−1.4 (3)
C11—O2—C10—O1	−1.0 (3)	C20—C19—C24—C23	1.7 (3)
C11—O2—C10—C6	179.33 (17)	C18—C19—C24—C23	−178.30 (19)
C7—C6—C10—O1	7.9 (3)	C22—C23—C24—C19	0.2 (3)
C5—C6—C10—O1	−168.6 (2)		

### Hydrogen-bond geometry (Å, º)

**Table:**

*D*—H···*A*	*D*—H	H···*A*	*D*···*A*	*D*—H···*A*
C1—H1···O3	0.95	2.26	2.853 (3)	120
C4—H4···O2	0.95	2.38	2.927 (2)	116
C21—H21···O3^i^	0.95	2.54	3.399 (3)	149
C2—H2···O1^ii^	0.95	2.63	3.531 (4)	157
C15—H15···O3^iii^	0.95	2.76	3.519 (4)	137
C1—H1···C15^ii^	0.95	2.74	3.6064 (3)	152
C11—H11*A*···C5^iv^	0.98	2.74	3.4906 (1)	133
C11—H11*B*···F1^v^	0.98	2.67	3.0585 (3)	104
C23—H23···O3^vi^	0.95	2.67	3.4875 (3)	143

Symmetry codes: (i) −*x*+3/2, *y*+1/2, −*z*+1/2; (ii) *x*, *y*−1, *z*; (iii) *x*, *y*+1, *z*; (iv) −*x*, −*y*+1, −*z*; (v) *x*−1/2, −*y*+3/2, *z*−1/2; (vi) −*x*+1/2, *y*+1/2, −*z*+1/2.
